# Materials for Powder-Based AC-Electroluminescence

**DOI:** 10.3390/ma3021353

**Published:** 2010-02-23

**Authors:** Michael Bredol, Hubert Schulze Dieckhoff

**Affiliations:** Department of Chemical Engineering, Fachhochschule Münster (University of Applied Sciences), Stegerwaldstraße 39, 48565 Steinfurt, Germany

**Keywords:** ZnS, AC-EL, phosphors, dielectrics

## Abstract

At present, thick film (powder based) alternating current electroluminescence (AC-EL) is the only technology available for the fabrication of large area, laterally structured and coloured light sources by simple printing techniques. Substrates for printing may be based on flexible polymers or glass, so the final devices can take up a huge variety of shapes. After an introduction of the underlying physics and chemistry, the review highlights the technical progress behind this development, concentrating on luminescent and dielectric materials used. Limitations of the available materials as well as room for further improvement are also discussed.

## 1. Introduction

Alternating current electroluminescence (AC-EL) in ZnS powder layers was discovered as early as 1936 by George Destriau [1] when working on the conductivity of certain metal alloys in the laboratory of Marie Curie in Paris. He immediately recognized their potential as a convenient light source and later developed the effect to application in AC-electroluminescent foils. However, the low light output and the limited durability of these first systems prevented widespread use of the original concept. Moreover, it took some time until the 1950s to develop a basic understanding of the new effect [2,3]; among others *Piper* and *Williams* started to establish first theoretical concepts [4]. Over the course of time, other electroluminescence variants have been described and investigated, namely DC-powder luminescence, AC-EL with evaporated or sputtered thin ZnS films (TFACEL) or DC-EL with thin films (injection luminescence). The overview of *Chadha* in [5] over “Powder Electroluminescence” gives a nice introduction into all these approaches, describing advantages and disadvantages. Among all these variants, TFACEL was commercially the most successful one, especially for pixelated alphanumeric displays. For lighting purposes with focus on high efficiency and satisfying colour rendering, none of these approaches were successful, and thus DC-electroluminescene in p/n-junctions of semiconductors (based on GaN alloys) has become the mainstream application of electroluminescence in lighting today.

The subject of this review will be powder-based thick film AC-EL. It got a first push to application between 1950 and 1963, but then soon was left behind by the emerging thin film techniques or DC variants [6]. Nevertheless, a second push occurred after the development of more durable phosphors around 1990, and thus powder-based thick-film AC-EL still is present in the market, since it is the only mature technology for flat and flexible large area light sources, at least as long as organic light emitting diodes (OLEDs) fail to deliver in this respect. Durability and light output of AC-electroluminescent foil lamps have made tremendous advances, mostly due to the improvements in the luminescent materials used. Somewhat surprising, these materials are still based more or less exclusively on ZnS, although a lot of effort has been invested into broadening the materials basis. Even more surprising is the fact, that a full theoretical understanding of the underlying physics is still missing; development of phosphors therefore is still based on trial and error approaches. Nevertheless, thick-film (powder) AC-EL in foil lamps today is applied for back-lighting of LCD displays or mobile phone keyboards, in automotive dashboards, for indoor illumination at modest light levels (e.g., luminescent tiles), safety installations, advertising purposes or decoration, thus mostly under conditions with low ambient light influx.

Powder electroluminescence from thick films is a profoundly different approach to light generation than TFACEL. While the latter is based on a (sometimes multiple) stack of semiconducting and dielectric layers deposited in a well-defined way by evaporation, sputtering or atomic layer epitaxy (ALE), the former has a more irregular structure with binder phases in its emissive layer as well as in dielectrics and electrodes. Main advantage however is the possibility to prepare most of theses layers by simple screen printing techniques (with the exemption of the necessary transparent electrode). For some time, a thick-film variant driven by direct current (DC) was developed as well, but could never really enter the mass market, partially because ZnS is a poor conductor and can not carry enough current to generate sufficient brightness. Instead, a complicated process of coating with Cu_2_S and subsequent “forming” is necessary, as explained in detail by *Chadha* in [5]. The AC variant does not have this problem, since it is a purely capacitive electrical load, and charges thus do not need to be transported over extended distances.

Although there are several mature commercial solutions based on foil lamps existing on the market (among others LCD backlighting, decorative articles, advertising), there is still a wish list for improvements of thick-film AC-EL, following current trends for the application of such light sources:

- Smaller phosphor particles for better printability, thinner stacks and smaller driving voltages
- Fully transparent systems
- Higher luminous output and total efficiency
- New transparent electrodes in order to save or replace expensive indium tin oxide (ITO)
- Less degradation, higher durability, introduction of new encapsulation approaches or humidity barriers and getters
- New phosphors for more colourful systems
- More simple (single-layer) structures, also high-temperature resistant

Whether it is possible to contribute at least to some of these issues will be discussed in the following sections.

## 2. Structure of AC-EL Foil Lamps

**Figure 1:**
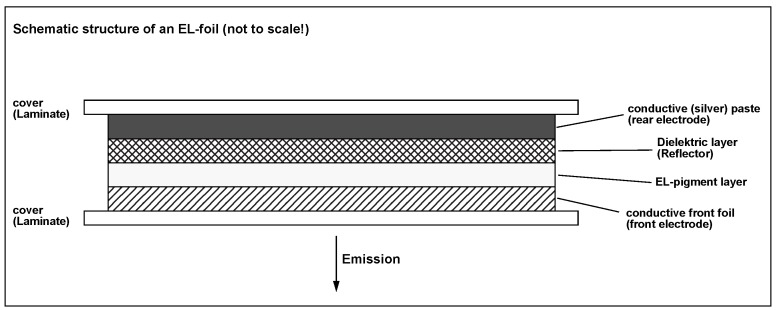
Internal structure (schematic) of an AC-EL foil lamp.

Powder AC-EL lamps have a very simple, printable internal structure. Figure 1 shows the essential elements, although not to scale. The transparent front electrode typically is made from polymer foil or glass sputtered with ITO; this layer is the only one, that is not regularly printed. However, the internal structure especially of the emitting layer is more complicated than depicted in Figure 1, since it contains (coated) phosphor particles as well as the components of a binder phase, typically an organic polymer with sufficient dielectric constant. In contrast to TFACEL, the individual grains therefore are insulated from each other, and thus there can be no volume currents in the active layer. The continuous semiconducting entities are typically as small as 10–20 *μ*m in all spatial directions, whereas in TFACEL devices, the two lateral dimensions may be very large, whereas the one perpendicular to the substrate is very thin. All electronic effects (carrier generation, carrier transport, luminescence) in thick-film AC-EL thus have to take place inside the individual grains of the powder layer; electrically they will carry the signature of (damped) displacement currents.

**Figure 2:**
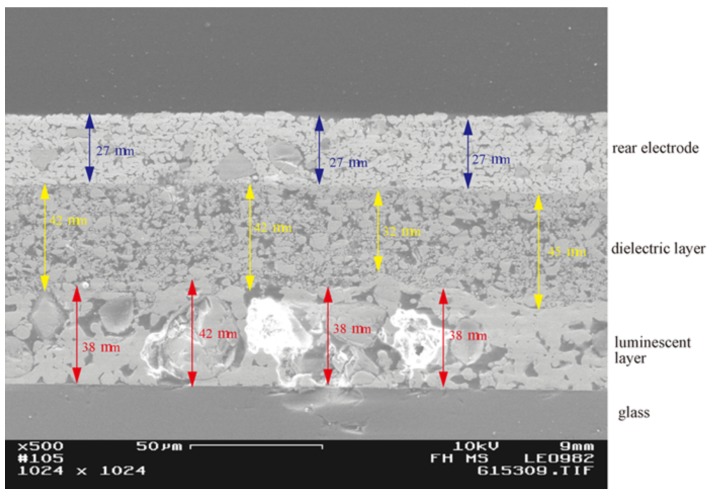
Electron micrograph of a cross section through a printed thick-film AC-EL stack.

Figure 2 shows the electron micrograph of a cross section through an experimental, printed thick-film stack with silver back electrode, BaTiO_3_ dielectric and luminescent particles embedded in an enamel. The dielectric layer is thicker than usual, but its granular structure is well displayed. The emissive layer shows even larger particles with a broader distribution of binder and particle sizes. The typical problem is clearly visible: the phosphor particles are essentially nearly as thick as the emissive layer, and the packing density of the phosphor in this layer is small and irregular. Moreover, the thickness of the luminescent layer can not be reduced and thus limits the total thickness of the whole system; transparency with such large scattering particles (if desired) will be impossible.

## 3. Physical Background

**Figure 3:**
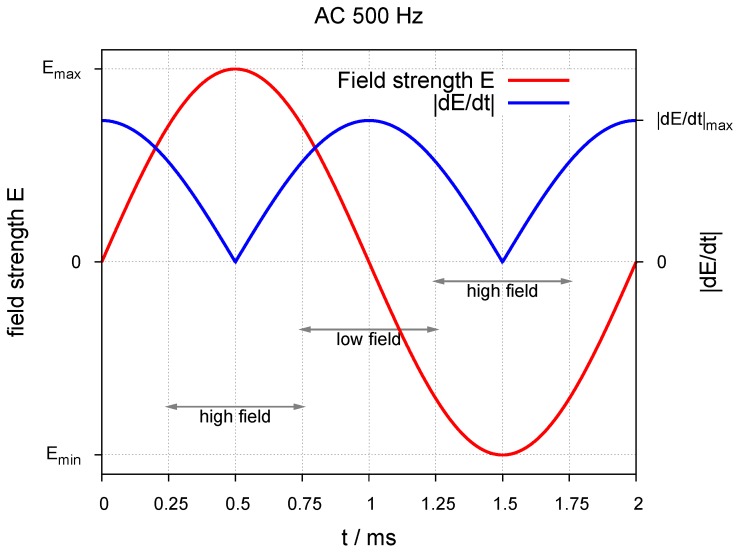
Time slots in an AC-El cell under typical excitation conditions.

In most cases, the driving voltage in thick-film AC-EL is alternated at a frequency between 50 and 1000 Hz. Figure 3 shows the time slots available for excitation and emission under typical excitation conditions of a 500 Hz sine wave: high and low fields are defining intervals, in which either excitation is possible or emission takes place. In general, higher frequencies and curve forms with a sharper rise and fall (e.g., square waves) are leading to faster degradation and thus are avoided, when possible, although the light output in most cases increases. The length of the time slots is large enough to allow for donor/acceptor-type luminescence with decay times in the ns-range; center luminescence with life times of more than 0.5 ms on the other hand does not match the slots and thus will get out of phase of the excitation cycles. Thick-film devices will only operate at voltages well above 50 V and thus may create a safety problem, especially on large area displays, where the necessary inverter has to deliver substantial power to the system.

Recent investigations [7] have shown, that light emission is predominantly originating from regions near the particle surface, concentrated in small spots. The number of these spots increases with increasing driving voltage (thus electrical field), and the emitting surface is switching from one side of the crystal to the other one under polarity reversal. These results support the idea, that electrons and holes are separated at the highest field strengths close to the surface and subsequently recombine radiatively on reversal of the polarity. However, such a mechanism needs imperfections (perhaps on the basis of needle-like Cu_2_S inclusions as suggested already by *Maeda* [8] back in 1958), facilitating carrier separation by disturbance of the applied field not only at the surface, but possibly also in the crystal volume. An ideal ZnS crystal would need much higher field strengths for charge carrier generation in an electrical field, than realized in working thick-film devices. Another potential source of imperfections are incomplete sphalerite/wurtzite transitions by repeated heating and cooling cycles over the transition temperature [9,10] (see also the section about ZnS preparation). Early reports about luminous “comets” [11] in the particles could never be supported in more recent investigations with higher microscopic resolution, using smaller emitting particles, and seem to be artifacts of the large crystals used in those preliminary studies.

For excitation, not the field strength across the whole device, but rather at the position of the individual ZnS grains is important. With a matrix with high dielectric constant, the external field can be “concentrated” on the embedded particles. The resulting local field strength can be calculated for spherical grains with relative dielectric constant $\epsilon_{r1}$ in a surrounding matrix with the dielectric constant $\epsilon_{r2}$ (Equation 1) with $E_{m}=U/d$ as mean field, *ϕ* as the volume fraction of the ZnS grains).

(1) $$E_{ZnS}=E_{m}\left[\frac{3\epsilon_{r2}}{2\epsilon_{r2}+\epsilon_{r1}-\phi \left(\epsilon_{r1}-\epsilon_{r2}\right)}\right]$$

**Figure 4:**
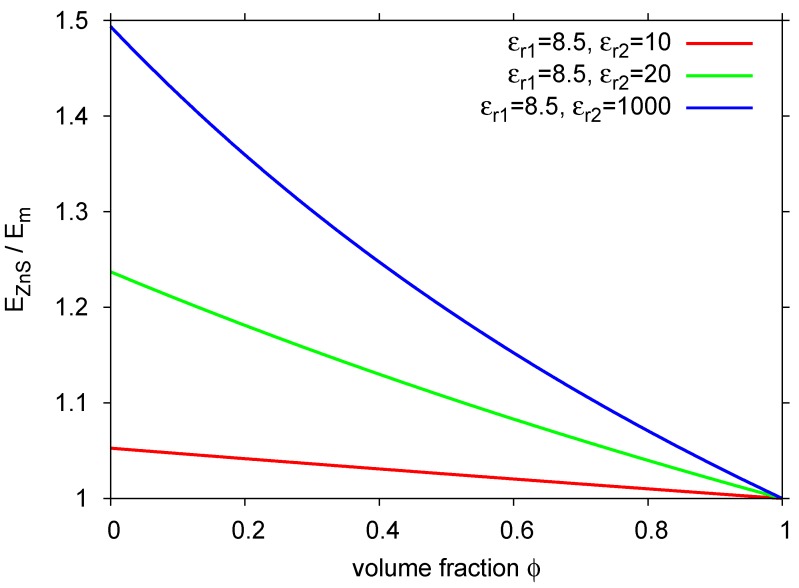
Electric field strength over luminescent particles.

Figure 4 shows, how a changing difference between dielectric constants of particle (8.5 at 2 MHz for ZnS) and matrix affects the local field strength. With typical thicknesses of the active layer of less than 50 μm and applied voltages of more than 100 V, electric field strengths are on the order of $10^{7}\;Vm^{-1}$. Similar reasoning holds for layer stacks: an insulating, reflecting layer as used in most devices should have a dielectric constant as high as possible in order to “concentrate” the field on the luminescent layer, which in turn should focus the field on the emitting particles.

Thick-film AC-El cells driven as a capacitive load show a dependence of brightness *B* on voltage *V*, that follows closely a quasi-linear curve as given by Equation 2, at least under practically useful conditions, with $V_{0}$ and $B_{0}$ as empirical constants.

(2) $$B=B_{0}\exp\left[-{\left(\frac{V_{0}}{V}\right)}^{1/2}\right]$$

**Figure 5:**
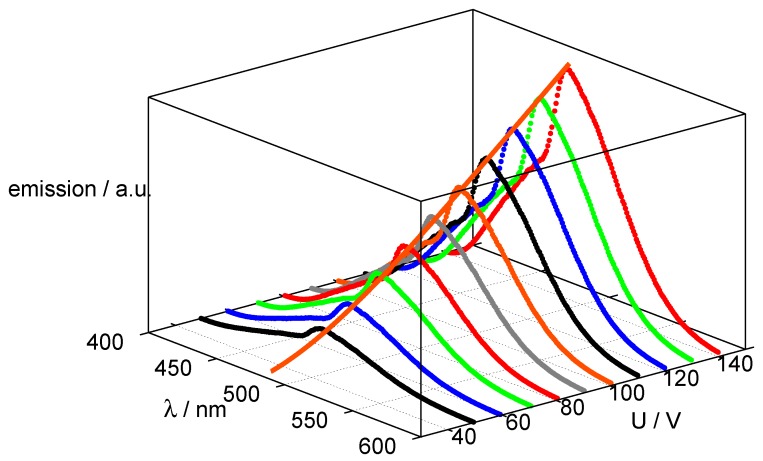
Emission spectra of conventional AC-El cell at various driving voltages, frequency: 300 Hz.

As an example from our own work, Figure 5 shows this behaviour for a conventional structure (as used also in foil lamps), screen-printed on conductive glass (sputtered with ITO) and driven *via* printed carbon back electrodes. The spectra do not change their shape with the driving voltage, but the peak intensity closely follows Equation 2.

**Figure 6:**
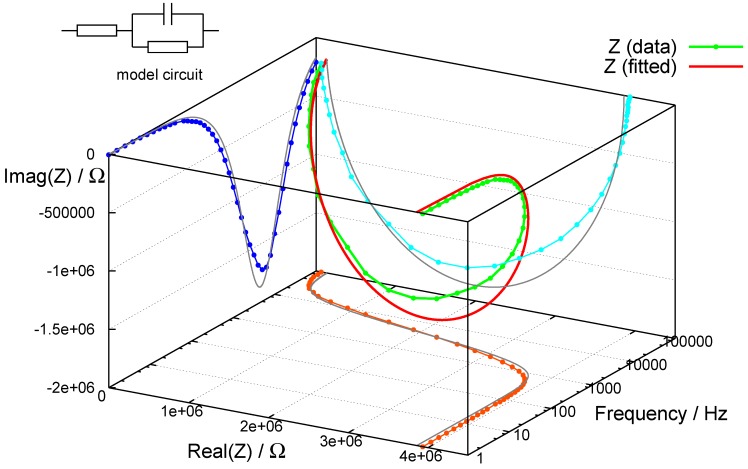
Impedance spectrum of a conventional AC-El cell at low voltage, together with fitted function.

The simplicity of the internal structure of a foil lamp is found back in its low-voltage (linear) impedance behaviour, which typically represents a capacitive load with series and shunt resistors in an equivalent circuit. Figure 6 shows a result from an experimental, screen-printed foil lamp as used also in Figure 5. (Complex) impedance Z(ω) and dielectric function $\epsilon^{*}\left(\omega \right)$ are connected by simple inversion ($C_{cell}$ is the capacitance of the empty measurement cell, ω=2πν, *i* is the imaginary unit):

(3) $$\epsilon^{*}\left(\omega \right)=\frac{1}{Z\left(\omega \right)i\omega C_{cell}}$$

Accordingly, the results of impedance spectroscopy can be represented also as a dielectric function and fitted to *Havriliak–Negami*’s equation for broadened dielectric relaxation ($\epsilon_{\infty }$ is the dielectric constant at very high frequency, $\epsilon_{stat}$ is the DC dielectric constant, *τ* is the relaxation time, $\delta_{0}$ describes DC conductivity, *α* and *β* are adjustable parameters) [12]:

(4) $$\epsilon^{*}\left(\omega \right)=\epsilon_{\infty }+\frac{\epsilon_{stat}-\epsilon_{\infty }}{{\left[1+{\left(i\omega \tau \right)}^{1-\alpha }\right]}^{\beta }}+i\frac{\delta_{0}}{\omega }$$

**Figure 7:**
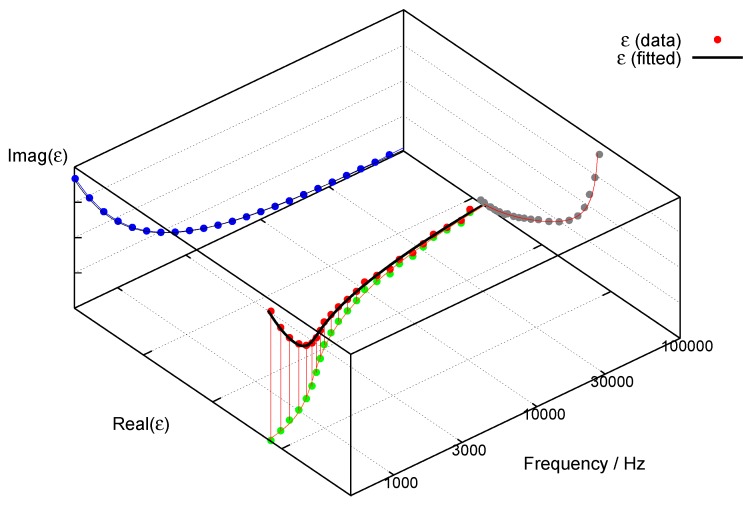
Dielectric function plotted from data in Figure 6, fitted to *Havriliak– Negami*’s equation.

Figure 7 shows a fraction of the same data as used for Figure 6, but as dielectric function with a fit to *Havriliak–Negami*’s equation. Figure 6 and Figure 7 depict the typical behaviour of a well-structured AC-EL thick film cell: small impedance at high frequency (dominated by the resistivity of the contacts) and very high impedance at small frequencies (limiting the loss by DC-conductivity). In both cases we note, that the validity of the data can be checked by application of the *Kramers-Kronig* relations; resonances occurring in the imaginary parts of *Z* or $\epsilon^{*}$ need to coincide with inflection points in the real parts. Of course, these exclusively diagnostic low-field impedance spectra shown here must not be confused with the (non-linear) impedance of the operating (emitting) device, driven under high-field-conditions. Equivalent circuits in this case have to contain strongly non-linear elements, typically back-to-back *Zener*-diodes. This has been worked out previously for the electrically more simple case of TFACEL [13]. However, power consumption still can only occur *via* displacement currents, since the total resistance of the device remains high. The complex displacement field $D^{*}$ can always be described as the answer to changing field conditions (but then with field-dependent dielectric constant):

(5) $$D^{*}\left(\omega ,t\right)=\epsilon^{*}\left(\omega ,E\right)\epsilon_{0}E^{*}\left(\omega ,t\right)$$

## 4. Materials for AC-Electroluminescence

### 4.1. Phosphors

#### ZnS

An excellent overview of the physical properties of ZnS for use in electroluminescent devices has been given by *Shepherd* and *Holloway* in a chapter of [14], although concentrating most of the discussion on thin-film-devices. All commercial devices are dominated by ZnS-phosphors (sometimes alloyed with ZnSe). Emission colours from blue to red are available, but the choice of colours is limited (ZnS:Cu,Cl emitting at 450 nm, ZnS:Cu,Al at 540 nm, ZnS:Cu,Mn,Al at 590 nm and ZnS:Cu at 690 nm), and it turned out to be difficult to tune them. In view of the attractive application as large area light source, especially the generation of stable white emission is a problem: either orange and blue emitting phosphors have to be mixed, or a blue or green emitter has to be combined with a fluorescent dye generating the necessary longer wavelengths [15]. In either case colour rendering of the light emitted is quite poor, but more severe is the problem, that the maintenance of different phosphors in blends tends to be different, and thus the colour of the emitted light will change gradually over the lifetime of the device. Therefore, careful selection of phosphors with high durability is necessary, and this problem has only been solved after the invention of rigidly encapsulated phosphors.

The general technology for production of ZnS phosphors is quite similar to the one used for ZnS phosphors for cathode ray tubes, which has been described in some detail in [16]. Basically, pure ZnS raw material is made in the first step by aqueous precipitation processes; care must be taken to purge the material from transition elements on the ppb scale. In a second step, dopant solutions (e.g., based on nitrates) are added to a dispersion of the raw ZnS in water. If possible dopants are deposited on the particles in this step by reaction with the sulfidic surface. This raw mixture then is dried carefully at low temperature to yield a dry powder, that then is fired in covered e.g., alumina or quartz crucibles. During the firing step, the atmosphere has to be controlled in order to prevent sulfur loss and oxidation. AC-EL powder phosphors are typically annealed just above the transition temperature between wurtzite and sphalerite form (ca. 1030 ${}^{\circ }$C) and then cooled under controlled conditions; recrystallization kinetics to sphalerite and the number of crystal defects established in this crucial step are very important for the final quality of the AC-EL powder phosphors. After cooling to room temperature, the material is gently milled for deagglomeration and then is ready for post-processing (e.g., application of a coating). A recent patent describes in detail, how comparably small and well-formed ZnS particles can be made, controlling precisely the individual steps of the general fabrication procedure [17]. One of the most comprehensive collections of general issues in context with phosphor preparation has been prepared by *Shionoya* and its successors [18] and should be consulted for further study.

Interestingly, all materials used nowadays for thick-film AC-EL contain Cu, which led several authors to the suggestion, that Cu_2_S-precipitates are involved in the excitation mechanism, forming tiny p/n-junction inside the emitting particles. However, such precipitates have never been found unambiguously, therefore their existence at least is quite doubtful. On the other hand, it has been found by EXAFS analysis of commercial, encapsulated phosphors, that the majority of Cu is not present on substitutional sites of the ZnS lattice, but rather in environments typical for CuS [19]. This led the authors to the conclusion, that a large part of the Cu is responsible for an excitation mechanism involving CuS-like clusters, and a smaller part then is acting as acceptor in subsequent donor-acceptor luminescence. Degradation then would be linked to depletion of the Cu-acceptors, possibly by electromigration to the surface or CuS-clusters. This has been supported by degradation studies under temperature variation, pointing to a diffusion–controlled underlying process [20]. A similar result with respect to localization of Cu has been found later in ZnS:Cu,Cl nanocrystals (size ca. 6 nm), explicitly concluding, that a siginificant amount of the Cu incorporated will be located in CuS–like structures on the nanoparticle surface [21]. On the contrary, other authors in a recent study report large solubility of Cu in ZnS, albeit in the presence of stacking faults, as visualized by HRTEM [22]. Stacking faults seem to be present in all efficient AC-EL phosphors, which has been confirmed very recently by the investigation of etch patterns [23]. On this background, it seems reasonable, that milling procedures play a crucial role in phosphor fabrication [24]. Nevertheless, after years of research, the relations of Cu, potential Cu-containing precipitates and stacking faults for thick-film AC-EL excitation are still far from being resolved.

Regardless of the details of the mechanism, excitation under AC-EL conditions leads to free electrons and holes. Under thin-film conditions electrons may be accelerated to very high velocities capable of direct (impact) excitation of luminescent centers. TFACEL thus is dominated by phosphors activated with Mn or lanthanide ions. Powder-based thick-film devices obviously do not offer this kind of energy transfer; field strengths are not high enough, and the active layer is not contiguous. Consequently, donor/acceptor-type luminescence is dominating such devices, with typical dopants like Cu, Ag, Au (acceptors) and Cl, Al (donors). ZnS:Mn is an exemption from this rule,since MnS is soluble up to large amounts on substitutional sites in the ZnS lattice, and Mn2+-ions can be excited efficiently by electron/hole-recombination. Therefore, the choice of phosphor systems for thick-film AC-EL is restricted: ZnS is needed as a host due to its unique possibilities of excitation in an alternating electrical field, and only donors and acceptors with levels close enough to the band edges of ZnS are candidates as luminescence activators. As a result, the number of efficient phosphors (with potentially more colours) essentially did not grow much after *Destriau*’s invention, and so we are still restricted to the available four or five colours for commercial applications.

The durability of ZnS phosphors depends partially on the stability of its complicated internal structure, but also very much on their interaction with the environment and the electrical driving conditions, with high frequencies and sharp polarity reversal being most detrimental influential factors. It is generally believed, that humidity penetrating the emitting layer leads to electrolytic decomposition of the ZnS particles, eventually generating free zinc metal (short-circuiting the emitting particle surface), explaining the often greyish colour after extended operation.

Although luminescence degradation obviously will have multiple causes, in most cases they convolute to a hyperbolical decay of the brightness *B* with time, as described by an empirical equation [25]:

(6) $$\frac{B}{B_{0}}={\left(1+\alpha t\right)}^{-1}$$

The constant *α* in Equation 6 is (among other factors) increasing with the driving frequency. With optimized phosphors (encapsulation against humidity) and driving conditions (moderate voltage, low frequency and sine waveform), life times of more than 3000 hrs at 200 $cd\;m^{-2}$ light output can be reached in foil lamps; at lower light levels life times can approach 10,000 hours. Meanwhile, manufacturers of foil lamps guarantee such values, even for outdoor use, provided the driving conditions are set appropriately. Low driving voltages and frequencies are also desired from the point of systems integration, e.g., in automotive environment. Other electronic components have to be shielded against dangerous high voltages and emitted electrical noise; suitable filters work best at low frequencies of the noise source. Other waveforms than sines will introduce high-frequency overtones and thus will not be tolerated in most technical environments.

Hermetic phosphor encapsulation was the main breakthrough in phosphor durability responsible for the now satisfying lifetimes of foil lamps. Key was the application of a completely dense coating of Al_2_O_3_ or SiO_2_ on the particles, which was accomplished by Chemical Vapour Deposition (CVD) in a fluidized bed (for more details, see section “coatings”). This proved to be feasible, because the ZnS particles employed are quite large (ca. 10–30 *μ*m). Current development activities, aiming at better printability, try to reduce the particle size well below 10 *μ*m, which on the other hand makes the application of high quality coatings with the established technologies more difficult. Therefore, practically, there seems to be a trade-off between device reliability and particle size; on the other hand particles as large as 30 *μ*m cause serious problems during the printing process. For well reproduced fine structures, printing pastes should contain much smaller particles, so this gap still has to be closed.

There are a few reports about electroluminescence from ZnS nanoparticles protected by organic ligands, e.g., from thin layers of pure nanoparticles [26] or nanorods prepared by aggregation [27,28], or under DC excitation of such layers [29,30]. Silicon nanoparticles embedded in silica (e.g., by ion implantation) represent a system of photoluminecent nanoparticles without any ligand sphere, and (at least weak) AC-EL has been observed in this case [31]. There are even reports about spatially directed emission from nanophosphors embedded in opals (photonic crystals) [32].

**Figure 8:**
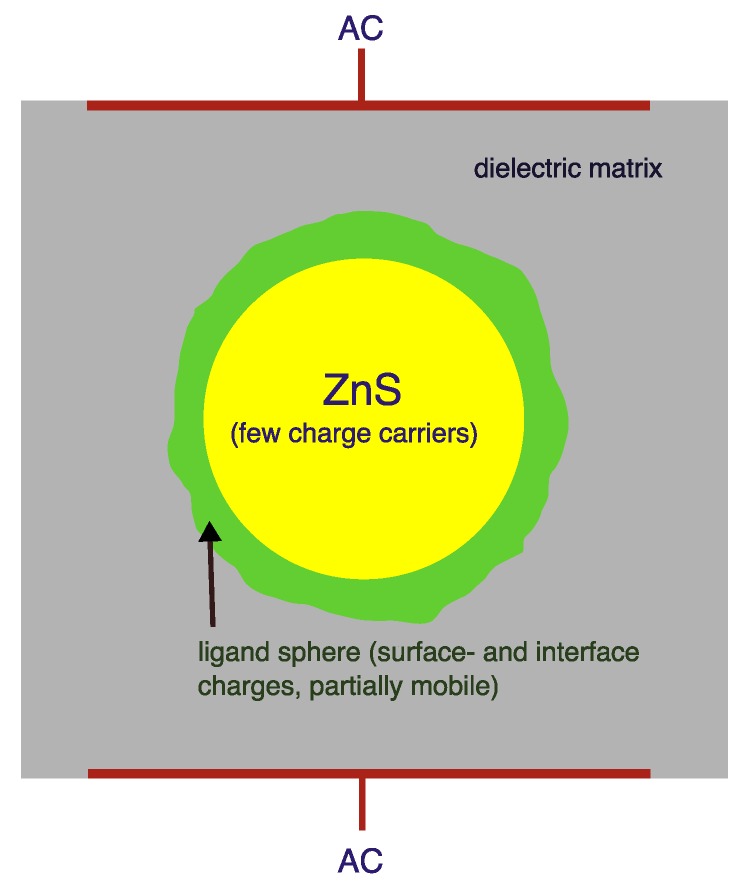
Schmatic of the electrical conditions in a ligand-stabilized nanoparticle.

However, nanoscaled materials in general failed to deliver meaningful AC-EL emission when embedded in printable polymers, although incorporation of photoluminescent ZnS:Mn-nanoparticles into dielectric polymers like PVDF has been demonstrated up to concentrations of more than 25% [33]. Most probably, this is due to the ligands necessary to stabilize and hydrophobize the nanoparticles; these necessarily very polar (and perhaps conductive) structures effectively reduce the local field over the nanoparticle, thus preventing excitation. Figure 8 depicts this in a simple model of a conductive layer around a poorly conducting core. A more successful approach to employ layers of nanosized emitting particles in this picture will need direct contact with charge injecting entities. Satoh *et al.* reported about experiments in this direction: they combined a thin (1.5 *μ*m) layer of e-beam evaporated ZnS:Mn with vertically aligned ZnO nanorods; the whole system was then packaged between two dielectric layers and metal and ITO electrodes [34]. Comparably low threshold voltages for emission were realized. Wood *et al.* recently demonstrated AC-EL emission from completely transparent stacks of spin-cast ZnS/ZnS:Mn nanoparticles and sputtered ZnS layers, also between two dielectric barriers [35]. Whether such approaches (actually being more comparable to TFACEL than to thick-film AC-EL) and their combinations can be transformed to thick-film approaches with printed emitting layers on large areas, remains open for further development. Especially the use of anisotropic ZnS structures (textured nanorods, nanobelts, *etc.*) might lead to new insight; approaches to fabricate such structures are already established on the research scene [36,37,38]. Since stacking faults were identified to play an important role in micron-sized crystals, new concepts to control stacking behaviour in ZnS-nanocrystals might also lead to novel materials with nanometer-sized particles [39].

An intermediate case between printed thick films and evaporated thin film devices are layers deposited from solution by dipping techniques. Thicknesses of the active layer then are on the order of 1–2 *μ*m, and the total stack can be constructed conventionally with conducting glass plates, active layer, insulating layer and metal electrode, as demonstrated for (Zn,Cd)S:Cu,F films [40]. The brightness of such devices follows Equation 2 and demonstrates, that the underlying mechanism is similar to thick film devices. An alternative is deposition by CVD techniques, and also here ideas can be borrowed from work on thin-film devices, as described e.g., in [41]. Future developments thus might well mix approaches from TFACEL with vacuum.free deposition methods and printing techniques.

#### CaS and SrS

Alkaline earth sulfides are the only alternative to ZnS with demonstrated, reasonable thick-film AC-EL luminescence. As with ZnS, thick-film AC-EL seems to be coupled to the presence of Cu in CaS. However, in contrast to ZnS, there are much more reports about emission from lanthanide ions, e.g., CaS:Cu,Eu, CaS:Cu,Sm, CaS:Cu,Er or CaS:Cu,Nd, probably due to the higher solubility of lanthanide ions in alkaline earth sulfides. Especially in nano-ZnS, there are indications, that lanthanide ions are insoluble [42], and thus they may be present in larger crystals not on substitutional sites, but rather in the form of clusters or nanoprecipitates and thus inaccessible for excitation *via* electron/hole-pairs. Eu^3+^ ions seem to be an exemption from this rule; several papers report at least photoluminescence from ZnS:Eu [43,44]. For CaS:Cu,Er it has been shown, that the AC-EL-efficiency follows Equation 2, which points to a excitation mechanism shared with ZnS [45]. Similar behaviour has been found in SrS, BaS and (Sr,Ba)S. However, all these materials are much more reactive in contact with H_2_O than ZnS and thus show much faster degradation; none of them are used commercially in thick-film AC-EL devices, whereas SrS:Ce and Srs:Cu,Ag have found much interest for TFACEL, where they are embedded in protective stacks. Under such conditions and in these applications they are much better understood, and there are physical and analytical models available [46]. For ternary sulfides (also of interest in TFACEL), to our best knowledge there are no reports about powder-based thick-film AC-EL.

#### Coatings

As outlined above, thick-film AC-EL phosphor particles need a dedicated microstructure, as established at the firing and cooling stage. Therefore, any post-processing of the phosphors should avoid temperatures above 500 ${}^{\circ }$C, since at this temperature the components of ZnS start to become mobile; sulfur loss at the surface and recrystallization processes are then to be expected, potentially being harmful for the microstructure established previously. Unfortunately, most desirable “hard” coating materials are refractories and need very high sintering temperatures before they form dense layers, which are in turn needed to protect the phosphors against humidity. Coating thick-film AC-EL phosphors thus can only be successful, if low temperature deposition techniques are applied. There are numerous reports about inorganic coatings applied to nanoparticles or microcrystals of ZnS by wet chemical methods, also e.g., through sol-gel chemistry on thick-film AC-EL phosphors [47]. However, none of them led to the kind of hard encapsulation, that is needed for AC-EL phosphors; they all suffer either from processing temperatures harmful for the phosphor, or heterogeneous nucleation leading to porous coatings. Such limitations could only be overcome by gas phase deposition techniques at low pressure, using reactive precursors. State of the art today thus is, that only CVD proved to deliver homogeneous, dense protective films, as needed for phosphors with improved durability under harsh operating conditions.

In order to be able to coat each individual phosphor particle, fluidized bed reactors turned out to be the most advantageous ones, but this technique requires comparable large (heavy) particles. Therefore, only comparably large phosphor particles, encapsulated by Al_2_O_3_ or SiO_2_, are yielding foil lamps with excellent durability, even under outdoor conditions. These developments have been described more or less exclusively in the patent literature, starting at about 1985 with claims filed by GTE [48], followed by more detailed procedures in the following five to six years.

A specialized process to prevent degradation through a coating with SiO_2_ in a fluidized bed reactor, using silane precursors and oxygen or water, then has been described in 1987 [49]. Two years later, a process employing aluminum alcoholate and water, leading to Al_2_O_3_-coatings, has been disclosed [50]. Soon thereafter, a variant using aluminum alkyls has been proposed as well [51]. The superior quality of powder AC-EL lamps fabricated from such phosphors was claimed in 1991 [52]. Later, these basic inventions have been improved in several details or have been modified with respect to precursors and procedures, but the method essentially remains always the same: comparably large phosphor particles in a fluidized or agitated bed are reacted with oxide precursors from the gas phase to generate thin, transparent, conformal coatings.

Fluidized bed reactors can be extremely versatile, since they can be extended into multistage units, capable either of application of multiple shells of the same material, or shells of different materials [53]; coatings wth mixtures of SiO_2_ and Al_2_O_3_ therefore have been proposed as well.

Among potential alternative coating materials, diamond-like-carbon (DLC) seems to be most promising. It can be applied from the gas phase, may be very thin but chemically extremely durable; it is well known as an excellent diffusion barrier, even against hydrogen. Processing has to be performed in vacuum, using fixed or agitated beds for the phosphor, but typically from a low pressure plasma fed with (mostly unsaturated) hydrocarbons [54]. Such (non-equilibrium) plasmas do not heat the powder much, thus the phosphors to be coated are well protected against thermal degradation during the coating process.

When testing the quality of a coating around ZnS phosphors without the need to observe a full AC-EL lamp e.g in climate chambers over extended periods, chemical tests with AgNO_3_-solution or surface spectrometric measurements (typically X-ray photo electron spectroscopy, XPS) are applied. In both cases, the presence (and amount) of sulfur at the particle surface is investigated: reaction with Ag^+^-ions changes the particle colour from yellowish to grey or black, whereas XPS can even distinguish between the various oxidation states of sulfur, if present at the surface at all. An alternative is exposure of phosphors to concentrated hydrochloric acid; H_2_S generated under attack of the acid can easily be visualized by lead acetate paper. Encapsulated high quality phosphors will show no sulfur under either of these tests. Degradation tests on the final devices today are mostly performed under accelerated conditions (elevated temperature, humid atmosphere, full electrical load), since high quality foil lamps are designed to operate for more than 5000 hours.

Of course, there have been attempts to coat the phosphor particles with the preferred dielectric (BaTiO_3_) in order to prevent any loss of field strength and enhance luminous output [55], but the sol-gel method employed did not produce dense, reliable coatings, whereas BaTiO_3_ is difficult to deposit by CVD methods. Therefore, such coatings never were exploited commercially.

### 4.2. Dielectrics

#### BaTiO_3_

BaTiO_3_ has a long tradition as (ferroelectric) material with potentially extremely high *ϵ*-values (up to 2000–3000 at room temperature, up to 10,000 at ca. 400 K, the *Curie*-temperature of the ferroelectric phase). The material is available in a variety of qualities; typical particle sizes are on the order of 200–1000 nm. Ceramics and compacts fabricated from such precursors show clear dependencies on particle sizes (see [56,57] and references therein). The dielectric properties are far from being constant, since there are changes in the crystal structure at ca. 270 K and ca. 180 K. This leads (among other effects) to a pronounced peak in the dielectric constant at about 130 ${}^{\circ }$C. The dielectric behaviour of BaTiO_3_ therefore is extremely sensitive on the details of the methods of preparation, processing and final morphology. This is especially important, if very fine powders are used. For application in thick-film AC-EL, there is the additional requirement, that the powder can be worked into a printable paste that yields dense, uniform layers without thermal postprocessing. Using fine BaTiO_3_ powders, this can be achieved with commercial binder polymers and plasticizers; several printable pastes are available on the market. The action of the dielectric layer is on one hand protection against short circuits and arcing, and on the other hand action as a white reflector concentrating light output on the front side of the device. In order not to weaken the electrical field at the location of the emitting particles, the layer has to be as thin as possible and have a *ϵ*-value as high as possible.

Apart from traditional solid state methods, many alternative ways have been tried, often based on sol-gel-chemistry or decomposition of suited precursors. In most cases, these approaches were used either to prepare the material directly as a layer, or to control the particle size or morphology (see e.g., [58,59]). Very small particles with good properties in AC powder EL devices have also been made by combustion synthesis [60]. Other approaches include partial subsitution of Ba against Sr in order to improve temperature characteristics; such approaches can directly yield layers with *ϵ*-values up to ca. 500 [61]. However, there is still large dependence of the final properties on the details of preparation [62]. In summary, there seems to be no feasible replacement for BaTiO_3_-powder-based materials, as long as a dielectric printable layer is needed in the stack.

#### Single layer solutions

The dielectric layer as protective layer leads to foil lamps, that tolerate cuts, deformation and punctuation; decorative articles can even be cut by ordinary scissors. However, in principle, a thick-film AC-EL emissive system does need only one single layer, provided the total dielectric properties are still tolerable [63]. To this end, suitable binders need to incorporate the dielectric function, and phosphor particles need to be as small as possible. For instance, ferroelectric (BaTiO_3_?) nano-particles or structures could be incorporated, or the binder matrix should have $\epsilon_{r}$ as high as possible, and of course protection of the phosphors against moisture still needs to be guaranteed. Naturally, the total light output must not be compromised too much.

An elegant solution would be the use of polymeric binders with high *ϵ* values; apart from cyanoacrylates (which are quite difficult to print), a suitable candidate would be polyvinylidenedifluorid (PVDF), which itself can develop ferroelectric structures; dispersions of the polymer can be modified to printable systems. In composites with inorganic particles, it has been demonstrated, that *ϵ* remains as high as 50 around room temperature [64] and thus this polymer would be ideal as base for a composite with AC-EL phosphors. The same result could be reached with polymers containing small BaTiO_3_ particles [65].

**Figure 9:**
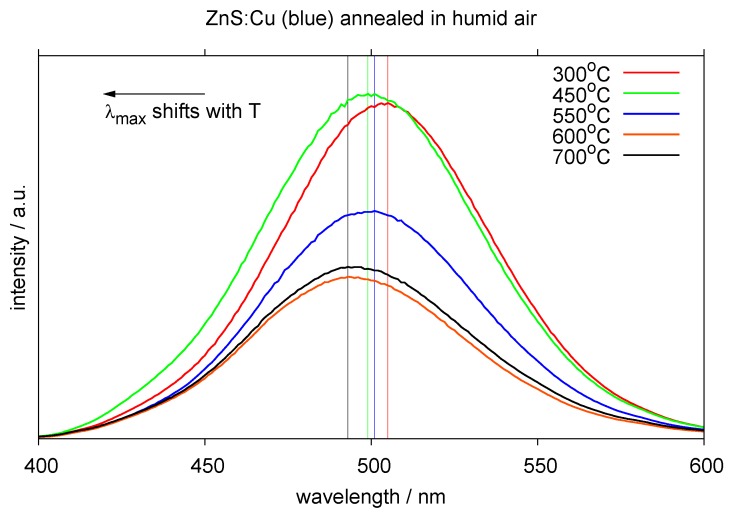
Spectral shift and reduction of emission intensity of AC-EL (ZnS:Cu) phosphor after heat treatment in humid air.

A special case is the use of high-temperature resistant dielectrics and fully inorganic AC-EL-structures. Such structures have been proposed already in the very early years of AC-electroluminescence (see e.g., [66,67,68] for an example with a dielectric enamel based on silica, titania and alkali / earth alkali oxides). They are desirable, because such structures may be deposited as a complete stack on glass, ready to be bent with the glass at 500–600 ${}^{\circ }$C to yield arbitrary forms based on glass. Improved resistance against corrosion offered by the enamel would be ideal for outdoor use, e.g., in automotive applications. A prerequisite for such solutions is the stability of the phosphors used against heat treatment during consolidation and bending. As outlined above, for ZnS-based phosphors, temperatures may be allowed to be not much higher than 500 ${}^{\circ }$C without compromising too much the light output (Figure 9 shows examples from our laboratory after prolonged heat treatment in humid atmosphere; the light output starts to decrease considerably beyond 500 ${}^{\circ }$C; the emission peak starts to shift to shorter wavelengths). Modern enamels offered commercially (e.g., by *Johnson-Matthey* or *Pemco*) are beginning to react at 600 ${}^{\circ }$C. For use as dielectric binder, the reaction temperature has to be reduced still in further developments.

**Figure 10:**
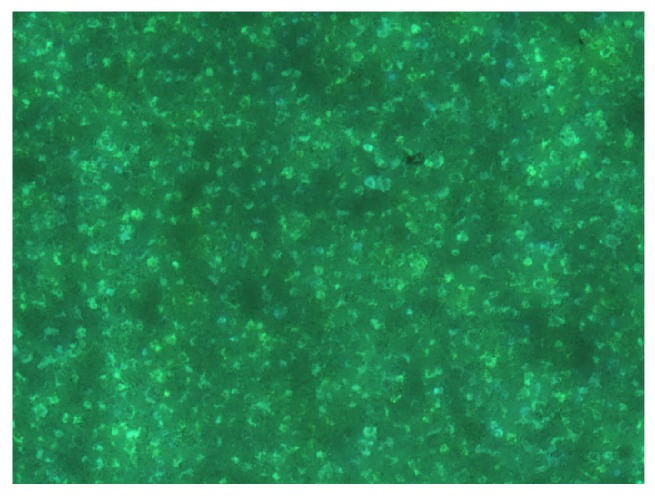
Fluorescence image of a cured (500 ${}^{\circ }$C) enamel/phosphor composite, taken through the substrate glass under UV illumination (366nm). Image width: 2 mm.

**Figure 11:**
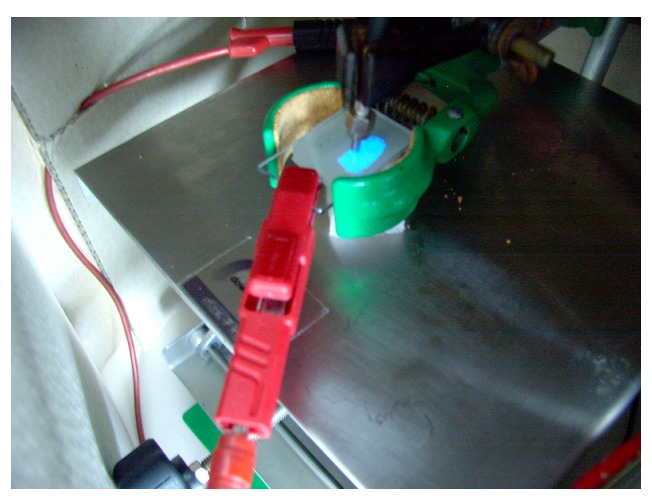
AC-luminescence after heat treatment at 500 ${}^{\circ }$C; light taken up by a fibre spectrometer.

**Figure 12:**
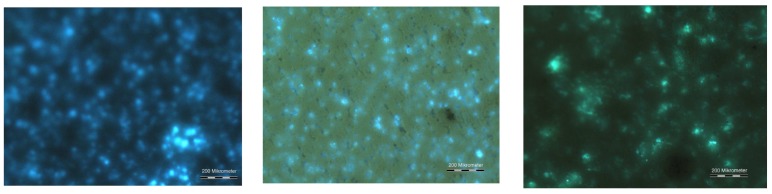
AC-luminescence after heat treatment, viewed through the substrate. Left: after 200 ${}^{\circ }$C. Center: after 500 ${}^{\circ }$C. Right: after 600 ${}^{\circ }$C. Scale bar: 200 *μ*m.

To illustrate the issues, we consider an experimental example. After mixing ZnS:Cu-phosphors with an enamel and a plasticizer (QPAC40), preparation of a layer by tape–casting on glass substrates and curing at 500 ${}^{\circ }$C, a fluorescence image taken through the substrate glass shows, that the phosphor particles are well dispersed and have retained their photoluminescent properties completely (Figure 10). AC–luminescence can also be observed, after heat treatment at 500 ${}^{\circ }$C of the complete stack over a C-back electrode (Figure 11). Figure 12 shows AC-EL emission through the substrate from a series with increasing temperature of the heat treatment; after treatment at 200 ${}^{\circ }$C, all phosphor particles are active (density of emitting points compared with Figure 10), whereas after treatment at 500 and 600 ${}^{\circ }$C more and more particles become inactive, either trough chemical attack by the components of the enamel, or by electrical short-circuiting.

A temperature of 600 ${}^{\circ }$C is already beyond the softening point of soda-lime-glass and thus allows for deformation of the substrate panel, which might be useful to prepare solid state preformed luminescent glass panels. If applied on large area, the enamel can be mixed with powdered high-*ϵ* (BaO-containing) glass. Such powders are available more or less for free from end-of-life CRT tubes (the front glass is rich in BaO and SrO as radiation shield); if necessary, they can be activated or adjusted in composition by steam processing [69].

### 4.3. Electrodes

Since the back electrode does not need to be transparent, metal powders or carbon black are printed onto the luminescent stack. Silver in this context shows the highest conductivity, but is expensive and tends to electromigration. Carbon on the other hand is cheap, but shows limitation due to its low conductivity. Aluminum paste might be an alternative but is difficult to process at low temperature. When reversing the stack (using solid metal as the back electrode), the flexibility of the system is lost. As a result, only silver and carbon are used in commercial applications. High conductivity is important for large areas, because the conductivity will limit the homogeneity of the electrical field over the active layer and thus the homogeneity of light emission. On small areas, carbon back electrodes are completely sufficient. For translucent devices the metal electrode can be printed in the form of thin stripes or grids; such an approach has been used to fabricate automotive roof windows that may change from diffusively transmitted daylight to AC-EL generated light during the night (in the more successful trials, a suited foil lamp was placed between two preformed glass sheets to form a compounded system [70]). There are also concepts with interdigitated metal electrodes [71].

ITO as transparent front electrode is also quite expensive, and it uses a limited resource (indium), that is concurrently used in large area displays and organic photovoltaic installations. Inks with nano-ITO-particles can be used to fabricate laterally structured front electrodes by printing techniques, and thus may reduce the consumption drastically (e.g., Evonik’s VP AdNano^®^ ITO is advertised for such purposes). Further alternatives based on Al-doped ZnO (AZO) [72], Sb-doped SnO_2_ (ATO) [73] or organics are under development for the transparent electrode, but will still need some time to replace ITO. A real alternative are conductive polymers like PEDOT:PSS (commercially available from various suppliers); they are easy to apply, may be neutral in colour and meanwhile show conductivities high enough to support AC-EL at least on moderately sized panels.

With the advent of graphene and graphene oxide as electronic materials, completely new solutions to the electrode systems may be possible in the future. Meanwhile, there are many chemical ways to variants of graphenes [74], and first working transparent electrodes have been made on the basis of graphene oxide, e.g., in solar cells [75]. Since a lot of work now is going into aqueous graphene dispersions of various kinds, novel printable transparent electrodes may eventually be available.

## 5. Conclusions

Powder-based thick-film AC-EL with technically interesting efficiency and durability seems to be coupled exclusively to encapsulated ZnS:Cu-derived phosphors. In the absence of a complete understanding of the underlying mechanism, further improvements therefore are not so much expected from new materials or slightly varied particle sizes, but rather from novel morphologies of the phosphor systems already available. However, the primary particle size must not be too small in order to retain the unique excitation mechanism in ZnS:Cu. Apart from ZnS-morphologies, new binders and dielectrics, especially if they are high-temperature resistant, may lead to new applictions of thick-film AC-EL not possible up to now.
